# Transcriptomic landscape of TIMP3 oncosuppressor activity in thyroid carcinoma

**DOI:** 10.1186/s12935-022-02811-8

**Published:** 2022-12-12

**Authors:** M. Mazzoni, K. Todoerti, L. Agnelli, E. Minna, S. Pagliardini, T. Di Marco, M. G. Borrello, A. Neri, A. Greco

**Affiliations:** 1 Molecular Mechanisms Unit, Research Department, Fondazione IRCCS Istituto Nazionale dei Tumori di Milano, Milan, Italy; 2 Department of Pathology and Laboratory Medicine, Fondazione IRCCS Istituto Nazionale dei Tumori di Milano, Milan, Italy; 3 Scientific Directorate, Azienda USL-IRCCS Reggio Emilia, Reggio Emilia, Italy

**Keywords:** TIMP3, Thyroid carcinoma, Inflammation, Gene expression

## Abstract

**Background:**

Papillary thyroid cancer (PTC) is the most frequent thyroid tumor. The tissue inhibitor of metalloproteinase-3 (TIMP3) gene encodes a matrix metalloproteinases inhibitor that exerts a tumor suppressor role in several tumor types. TIMP3 is frequently downregulated in PTC by promoter methylation. We have previously functionally demonstrated that TIMP3 exerts an oncosuppressor role in PTC: TIMP3 restoration in the PTC-derived NIM1 cell line affects in vitro migration, invasion and adhesive capability, while reduces tumor growth, angiogenesis and macrophage recruitment in vivo. To get a deeper insight on the mediators of TIMP3 oncosuppressor activity in thyroid tumors, here we focused on the TIMP3 related transcriptome.

**Methods:**

TCGA database was used for investigating the genes differentially expressed in PTC samples with low and high TIMP3 expression. Genome wide expression analysis of clones NIM1-T23 (expressing a high level of TIMP3 protein) and NIM1-EV (control empty vector) was performed. Gene sets and functional enrichment analysis with clusterProfiler were applied to identify the modulated biological processes and pathways. CIBERSORT was used to evaluate the distribution of different immunological cell types in TCGA-PTC tumor samples with different TIMP3 expression levels. Real time PCR was performed for the validation of selected genes.

**Results:**

Thyroid tumors with TIMP3-high expression showed a down-modulation of inflammation-related gene sets, along with a reduced protumoral hematopoietic cells fraction; an enrichment of cell adhesion functions was also identified. Similar results were obtained in the TIMP3-overexpessing NIM1 cells in vitro model, where a down-regulation of immune-related function gene sets, some of which also identified in tumor samples, was observed. Interestingly, through enrichment analysis, were also recognized terms related to cell adhesion, extracellular matrix organization, blood vessel maintenance and vascular process functions that have been found modulated in our previous in vitro and in vivo functional studies.

**Conclusions:**

Our results highlight the correlation of TIMP3 expression levels with the regulation of inflammatory functions and the immune infiltration composition associated with different PTC prognosis, thus providing a broader view on the oncosuppressor role of TIMP3 in PTC.

**Supplementary Information:**

The online version contains supplementary material available at 10.1186/s12935-022-02811-8.

## Introduction

Thyroid cancer is the most frequent endocrine neoplasia whose incidence is increased in the last decades [1]. The majority of thyroid cancers arise from follicular cells and it includes several histological types differing in biological and clinical features. Well-differentiated papillary (PTC) and follicular (FTC) carcinoma have generally a good prognosis, being treatable with surgery and radioiodine therapy; however, in some cases, they can progress to more aggressive, incurable forms. Poorly differentiated and anaplastic thyroid carcinoma (PDTC and ATC) are rare, associated to poor prognosis, with survival reduced to few months in the case of ATC [2].

PTC is the most frequent thyroid carcinoma, and it is characterized by highly prevalent and mutually exclusive genetic alterations along the RTK/RAS/BRAF/MAPK axis, with BRAFV600E mutation as the most frequent [3]. Novel, less frequent, PTC driver genetic alterations have been identified by recent integrated genomic analyses [4]. Methylation and consequent silencing of tumor suppressor genes is reported as common event in thyroid tumors. PTEN and RASSF1A have been found aberrantly methylated in both benign thyroid neoplasm and thyroid cancer [5, 6]. In PTC, TIMP3, DAPK, and RARβ2 methylation has been reported to be associated with aggressive pathological characteristics [7]. Silencing of several thyroid-specific genes via promoter methylation, including NIS, TSHR, SLC26A4 and SLC5A8, has also been recognized [7–9]. More recently, a comprehensive DNA methylation signature of tumor suppressor genes involved in thyroid neoplasia has been proposed [10, 11].

Tissue inhibitor of metalloproteinase-3 (TIMP3) gene, located on chromosome 22q12.1-q13.2, belongs to the four members TIMPs gene family which encode the inhibitors of the matrix metalloproteinases (MMPs), a group of peptidases involved in degradation of the extracellular matrix (ECM) [12]. The balance between MMPs and TIMPs activities is particularly important to ensure the integrity of the ECM, and any alteration of the latter may affect several biological processes, including carcinogenesis [12]. TIMP3 is a 24 kDa secreted glycoprotein; in contrast to the other members of TIMPs family, it exerts inhibitory activity on a wide range of ADAMs family proteins, including tumor necrosis factor-a (TNF-a) convertase (TACE (tumor necrosis factor-a-converting enzyme) and ADAM-17 (ADAM metallopeptidase domain 17) [13], and this inhibition is crucial for controlling TNF-mediated inflammation [14]. Beyond cancer, impairment of TIMP3 activity is involved in several human pathologies as cardiovascular diseases and retinopathies [15, 16].

TIMP3 exerts a tumor suppressor role in several tumor types, where it regulates proliferation, apoptosis, angiogenesis, migration and invasion [17–19]. TIMP3 gene undergoes silencing through several different mechanisms. Loss or downregulation of TIMP3 expression by hypermethylation of the gene promoter region has been reported in several tumor types, including ovarian cancer, head and neck carcinoma and esophageal adenocarcinoma [20–22]. Post-transcriptional regulation of TIMP3 by miRNAs, such as miR-21, miR-221, miR-222 and miR-373 has been also widely recognized in different tumors [23–25]. TIMP3 is considered a potential therapeutic target for cancer; studies focusing on normalizing or reactivating the expression of TIMP3 as a potential anticancer therapy have been recently proposed [26].

In thyroid tumors TIMP3 methylation, and hence its downregulation, has been reported [27] and also found significantly associated with several aggressive features of PTC, including extrathyroidal invasion, lymph node metastasis, multifocality, advanced tumor stages and BRAFV600E mutation [7]. Furthermore, as reported in other tumor types, recent evidences suggest that TIMP3 suppression in thyroid cancer may be related also to miRNA deregulation [28, 29].

In a previous work we demonstrated that TIMP3 exerts an oncosuppressor role in thyroid carcinogenesis. In addition to confirming TIMP3 downregulation in a consistent fraction of PTC, we evaluated the functional consequences of TIMP3 retrieval in a PTC-derived cell line model. We found that TIMP3 restoration in NIM1 cell line, in which the expression of TIMP3 is silenced by promoter hypermethylation, reduced in vitro migration, invasion and anchorage-independent growth. Using a mouse xenograft model, we demonstrated that restoration of TIMP3 activity reduces tumor growth, concomitantly with reduction of angiogenesis and macrophage recruitment at tumor site [30]. Our previous findings clearly demonstrated a negative regulatory role of TIMP3 in thyroid cancer; however, the downstream effectors of TIMP3 in thyroid tumor cells are not known.

To have a broader view on the mediators of TIMP3 oncosuppressor activity in thyroid tumors, we focused on the TIMP3 related transcriptome. By interrogating the TCGA-PTC data set and performing a genome-wide expression analysis of TIMP3-restored NIM1 cells, we show that different expression levels of TIMP3 lead to transcriptional changes that modulate, among others, inflammatory functions.

## Results and discussion

### Analysis of thyroid cancer TCGA data

**Fig. 1:**
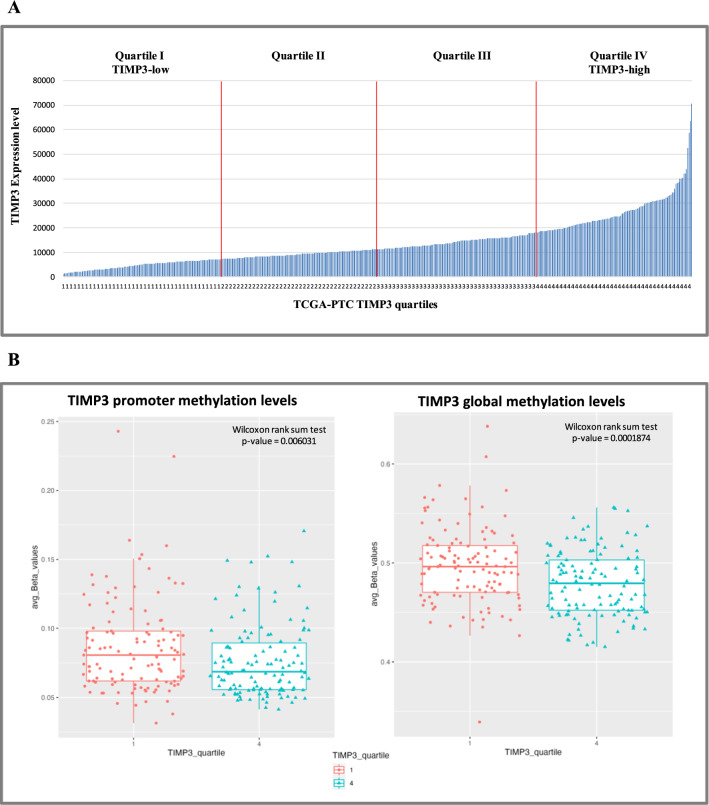
**A** TCGA-PTC tumors cohort stratified in four quartiles according to TIMP3 expression levels. **B** Methylation levels of TIMP3 gene in 121 quartile I and 122 quartile IV PTC cases. The average Beta values (y-axis) were calculated for each quartile (x-axis) on the four probes specific for the CpG Island nearer to TSS (left panel), and on all ten probes profiling TIMP3 global methylation status, also considering shore and shelf regions (right panel). Significant p-value of Wilcoxon rank sum test with continuity correction is reported for each analysis

We first analyzed the transcriptomic features that could be associated with TIMP3 level in thyroid cancer. To this aim we considered 486 thyroid tumors in the TCGA cohort [4] analyzed by RNA sequencing and for which clinical and molecular data were available. In particular, tumors were stratified in quartiles according to TIMP3 expression level, thus evidencing up to six fold-change (FC) reduced expression in quartile I compared to quartile IV, each including 122 PTC samples (Fig. 1A). With the aim of exploring which possible mechanisms could be involved in TIMP3 gene expression regulation, we examined TIMP3 methylation status and its correlation with gene expression levels in each quartile group. In particular, slightly lower methylation levels were observed in TIMP3 quartile IV in comparison to quartile I, by considering both the promoter/TSS and the global methylation status (Fig. 1B). The widest range of global methylation levels was observed in TIMP3 quartile I group, without reaching any significant correlation with gene expression (Additional file 1: Table S1). Otherwise, a moderate inverse correlation was observed in PTC cases showing higher TIMP3 expression levels, considering both TIMP3 promoter/TSS region and global methylation levels (Additional file 1: Table S1).

These observations suggest that other mechanisms may contribute to TIMP3 gene expression regulation. For this purpose, we investigated the possible correlation of TIMP3 expression levels with the expression of several miRNAs (miR-21, miR-221, miR-222, miR-372) targeting TIMP3, in each quartile group. In particular, a significant negative correlation has been observed with miR-21 expression levels (p-value = 0.002), in quartile I PTC cases, whereas no significant correlation was evidenced with any other tested miRNAs. On the contrary, no correlation was evidenced in TIMP3 quartile IV cases (Additional file 2: Table S2).

In accordance with literature data, in comparison with TIMP3-high (quartile IV), TIMP3-low (quartile I) cases presented a higher fraction of more advanced (III and IV) clinical stages (34% vs 24%, quartile I vs IV), a significant enrichment of BRAFV600E positive samples (63% vs 37%, p-value = 0.003) and of aggressive Tall-cell variant cases (12% vs 1%, p-value = 0.0045), and a less proportion of good prognosis conventional and follicular variants (Additional file 3: Table S3). No significant differences in overall survival were observed between 100 TIMP3 quartile IV and 106 quartile I cases, for which survival data were available in TCGA-THCA cohort (Additional file 4: Fig. S1).

**Fig. 2:**
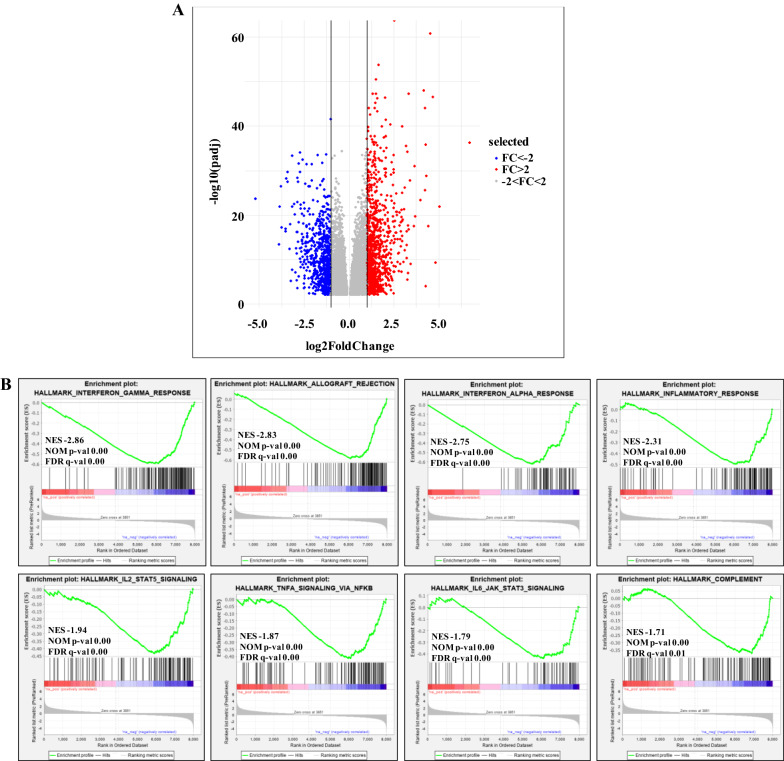
Differential gene expression results in TCGA papillary thyroid tumors, according to TIMP3 expression level. **A** Volcano Plot of the differentially expressed genes in TIMP3 IV versus I quartile at FDR 1%; blue and red dots show, respectively, 711 down-regulated and 949 up-regulated genes, with a FC of at least 2 in absolute value. **B** Enrichment plots of selected gene sets that were significantly down-regulated in high versus low TIMP3 expressing tumors by GSEA analysis. Normalized enrichment score (NES), nominal p-value (NOM p-val) and false discovery rate q-value (FDR q-val) are reported for each plot

**Fig. 3:**
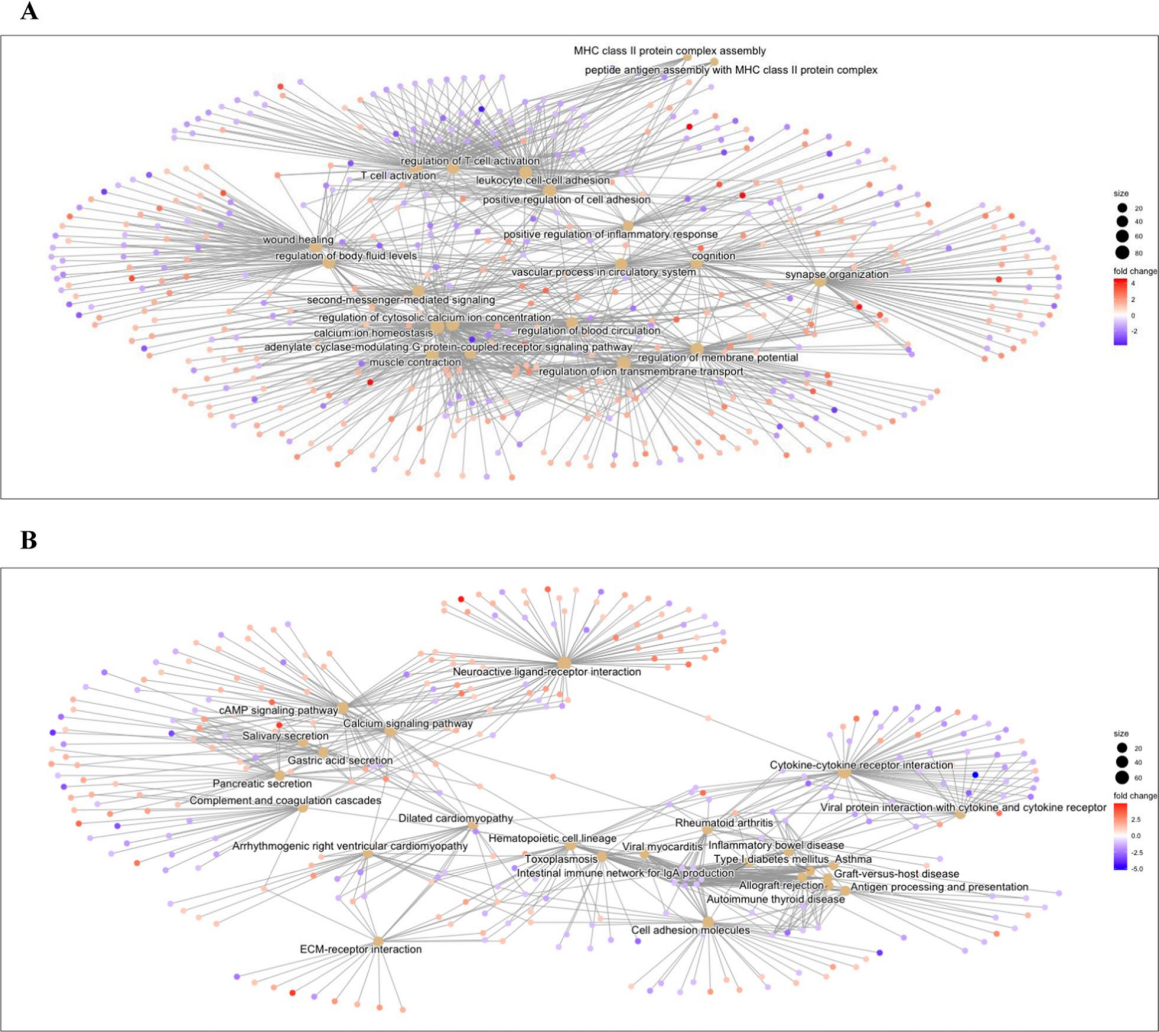
Enrichment analyses of differentially expressed genes between extreme TIMP3 quartiles of TCGA papillary thyroid tumors. **A** Cnetplot of top 20 enriched Gene Ontology Biological Process (GO-BP) terms in 1660 differentially expressed genes with at least 2 absolute FC. **B** Cnetplot of top 25 enriched KEGG terms in 1660 gene list

In order to define which genes were significantly modulated in association to TIMP3 expression level, we compared the global gene expression profiles of TIMP3-high versus TIMP3-low PTC samples (Fig. 2A evidences the most differentially expressed genes). To define the molecular pathways potentially modulated in association to TIMP3 expression, the dataset has been interrogated by Gene Set Enrichment Analysis (GSEA). Among the 16 significantly enriched Hallmark gene sets, 14 were down-regulated, whereas only two resulted positively modulated in TIMP3-high compared to TIMP3-low tumors (Additional file 5: Table S4). In particular, several cell cycle, cytokine signaling and inflammation pathway gene sets resulted under-expressed in samples with high TIMP3 expression level. The identification of several immune-related functions gene sets (Fig. 2B) indicates that TIMP3 capability to restrain inflammation is associated with transcriptional regulation of related gene sets. Moreover, Gene Ontology and KEGG enrichment analyses revealed the regulation of cell adhesion and inflammatory response terms (Additional file 6: Table S5 and Fig. 3A), as well as of pathways related to cell adhesion molecules, autoimmunity and inflammatory diseases/responses (Additional file 7: Table S6 and Fig. 3B).

**Fig. 4:**
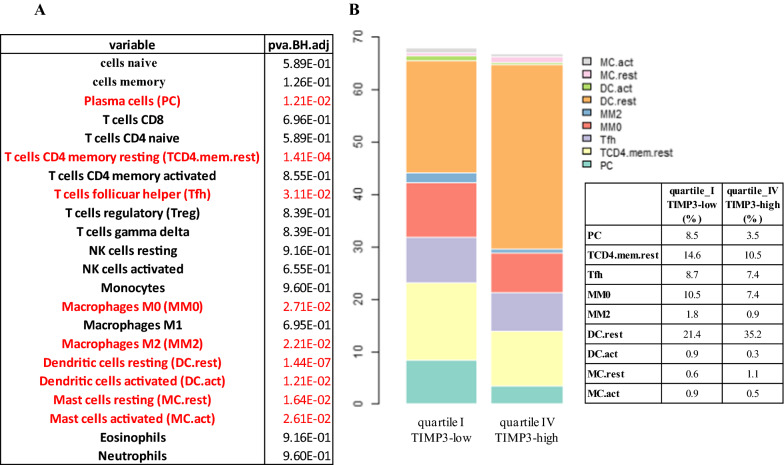
Immune cell deconvolution results using CIBERSORT on TCGA papillary thyroid tumors. **A** Wilcoxon test results comparing each LM22 component in TIMP3 quartile I vs IV. BH adjustment was applied. Significant p-values are highlighted in red. **B** Barplot of cell types from LM22 signature that were differentially represented by CIBERSORT deconvolution analysis in TIMP3 quartile I in comparison to quartile IV in TCGA-PTC samples. Relative percentages are reported for significantly enriched cell types in each group

Based on the recurrent presence of terms related to inflammation emerging by both kinds of enrichment analyses, we further explored the immune cell subsets differently represented in tumors with distinct TIMP3 expression levels. By CIBERSORT algorithm, we assessed the relative proportions of different immunological cell types in TIMP3-high and TIMP3-low PTC samples**.** Among 22 human hematopoietic cell phenotypes, 9 resulted differently distributed between the two TIMP3 groups (Fig. 4A). A significant enrichment of T cells CD4 memory resting, Macrophage M0, T cells follicular helper and Plasma cell types was observed in TIMP3-low, whereas Dendritic cells resting showed the highest percentage in TIMP3-high samples. When considering less abundant cell types, Macrophage M2, Dendritic and Mast cells activated resulted significantly enriched in the TIMP3-low, while Mast cells resting were more represented in the TIMP3-high samples (Fig. 4A and B). These results suggest that different TIMP3 expression levels may influence the tumor immune landscape, affecting the proportion of different immune cell classes.

The role of inflammation in thyroid cancer is well known. Condition predisposing to cancer and causative genetic events trigger the establishment of an inflammatory microenvironment useful for tumor onset and progression. Thyroid associated oncogenes are able to induce a proinflammatory program that includes the production of molecules able to recruit and differentiate/polarize monocytes [31, 32]. Indeed, M2-like tumor associated macrophages (TAMs) infiltration is frequently detected in human thyroid tumors, is increased in advanced ones and facilitates tumor progression [33]. Our results suggest a correlation between TIMP3 deficiency and inflammation in PTC.

Among the enriched populations identified in TIMP3-low tumors, Macrophages M0, Macrophages M2 and dendritic and mast cells, known to exert a protumoral activity, have been already reported to correlate with worst thyroid cancer features. Using data derived from TCGA database Xie et al. reported that, compared with normal tissues, PTC had higher overall immune cells level, with a significant increasing in the abundance and proportion of tumor-promoting immune cells during the occurrence and development of PTC. Indeed, the high-immunity group exhibited more advanced stages, larger tumor sizes, greater lymph node metastases, higher tall cell tumors and more BRAFV600E mutations [34]. Analogous results were reported by Jia et al. [35]. Bergdorf et al. confirmed the presence of certain immune cell types in PTC, particularly dendritic cells and neutrophils, strongly correlated with histological subtypes, mutational status, tumor stage and lymph node metastases [36]. Our data are in agreement with other studies, being TIMP3 low-expression itself correlated with worst PTC prognosis.

Our results suggest that TIMP3 loss plays a role in triggering the immune infiltration associated with worst PTC prognosis. Thus, TIMP3 oncosuppressor role in PTC involves, among other, a tight regulation of protumoral microenvironment.

### Global gene expression analysis of TIMP3-restored NIM1 cells

To investigate a direct link between TIMP3 expression and the modulated gene sets detected in tumor samples with different TIMP3 expression levels, we took advantage of an in vitro model available in our laboratory. In the context of our previous work, we restored TIMP3 expression in the PTC-derived NIM1 cell line, in which the expression of TIMP3 is silenced by promoter hypermethylation. By cDNA transfection we produced three stable clones expressing exogenous TIMP3, named NIM-T. TIMP3 restoration reduced in vitro migration, invasion and anchorage-independent growth of NIM1 cells; moreover, NIM-T clones displayed diminished in vivo tumorigenicity, concomitantly with reduction of angiogenesis and macrophage infiltration [30].

**Fig. 5:**
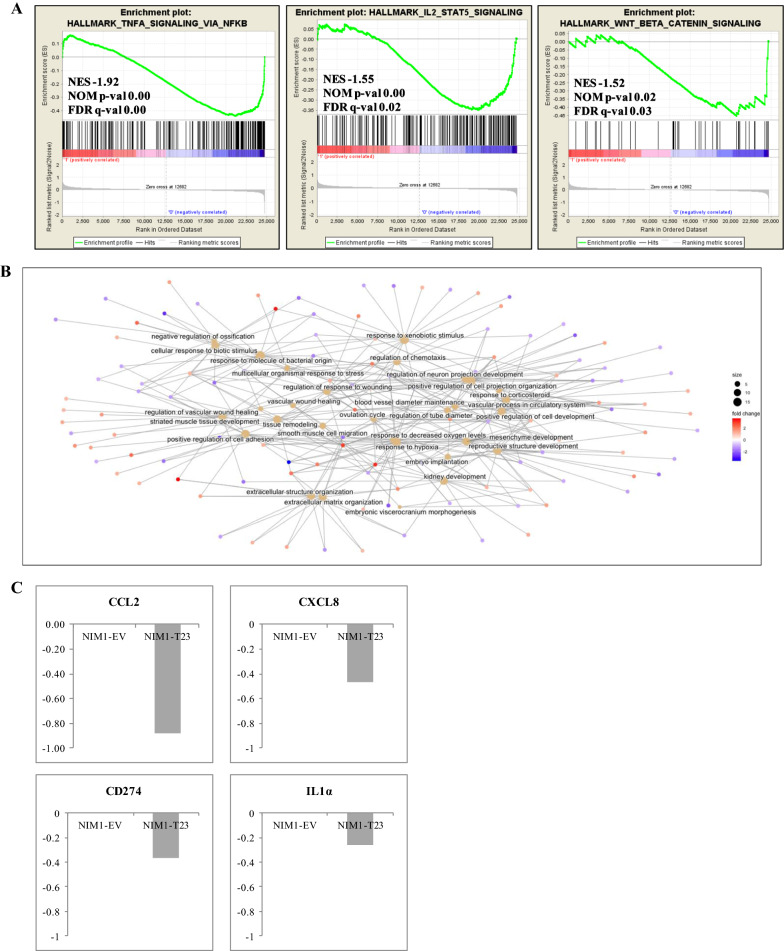
Gene expression profiling of NIM1-TIMP3 clone. **A** Enrichment plots of selected gene sets that were significantly down-regulated in NIM-T23 compared to control NIM1-EV cells. NES, NOM p-value and FDR q-value are reported for each plot. **B** Cnetplot of top 30 GO-BP terms enriched in 290 differentially expressed genes with a FC of at least 2 in absolute value in NIM1-T23 cells. **C** qRT-PCR analysis of CCL2, CXCL8, CD274 and IL-1α genes with a customized TaqMan Low Density Array. Values are presented as relative quantity (RQ Log10) normalized for HPRT-1 housekeeping gene expression. Data are normalized as ratio to the corresponding control, considered NIM1-EV = 0 as baseline and represent the mean of two independent experiments

Here we performed a genome wide expression analysis for NIM1-T23 (expressing the highest level of TIMP3 protein) and NIM1-EV (control empty vector) clones, and the whole gene expression matrix (24731 annotated genes) was interrogated as well by functional enrichment analysis*.* All the seventeen Hallmark gene sets resulting significantly enriched in TIMP3 overexpressing cells were down-regulated (Additional file 8: Table S7), particularly involving cell cycle gene sets that were found under-expressed also in TIMP3-high PTC cases (Additional file 5: Table S4), together with signaling and metabolic pathways, DNA damage and development functions. Interestingly, among them, we found some immune-related function gene sets, like Wnt/β-catenin signaling, and those involving TNFα/NFKB and IL-2/STAT5 pathways, both down-regulated even in PTC cases highly expressing TIMP3 (Figs. 2B and 5A). The regulation of the above pathways by TIMP3 is well known, and ultimately it results in the regulation of inflammation. Indeed, several authors have reported the development of inflammatory conditions as consequence of TIMP3 deficiency, in close association to enhanced TNFα release [14, 37], along with an increase of inflammatory cells infiltration and cytokines expression [37]. An increase of β-catenin signaling in TIMP3 deficient in vitro model has also been recognized [39]. Notably, NF-κB plays a critical role in regulating survival, activation and differentiation of innate immune cells (including macrophages, dendritic cells and neutrophils) and inflammatory T cells [40]; IL-2/STAT5 signaling pathway is particularly relevant in the regulation of T cell development and function [41], while Wnt/β-catenin pathway has been recently described to have a role in regulating immune cell infiltration of the tumor microenvironment [42]. The results obtained with the in vitro model corroborate the notion that the inflammation transcriptional program detected in human tumors is directly triggered by TIMP3 expression. Indeed, interestingly, the TNFα/NFKB and IL-2/STAT5 gene sets resulted down-regulated in TIMP3 overexpression condition, both in the in vitro model (Additional file 8: Table S7 and Fig. 5A) and in TCGA TIMP3-high tumors (Additional file 5: Table S4 and Fig. 2B). Moreover, these findings are also in line with the significant enrichment of macrophages and T cell types that was identified in the group of tumors at the lowest TIMP3 expression, by CIBERSORT analysis in TCGA tumors.

Supervised gene expression analysis in NIM1-T23 clone compared to NIM1-EV control allowed the identification of 290 differentially expressed genes (Additional file 9: Table S8), 157 of which (54%) were up-regulated including, as expected, TIMP3 gene among the top overexpressed (FC = 20.6). Particularly, among the relevant enrichments evidenced by clusterProfiler analysis, several were recognized related to cell adhesion, extracellular matrix organization, blood vessel maintenance and vascular process (Additional file 10: Table S9 and Fig. 5B); of note, all these functions have been found modulated in our previous in vitro and in vivo functional studies [28]. Moreover, several terms related to inflammation (as response to molecule of bacterial origin and regulation of chemotaxis), were found enriched in differentially expressed genes (Additional file 10: Table S9 and Fig. 5B). Particularly, among these, *CCL2* (the main macrophages chemoattractant), *CXCL8* (chemotactic factor for neutrophils, basophils and T cells), *CD274* (coding for the immune inhibitory receptor ligand PD-L1) and *IL1α* (involved in B-cell maturation and proliferation) genes were validated by real time PCR analysis, which confirmed their downregulation in the presence of TIMP3 (Fig. 5C).

Overall these results suggest that, upon TIMP3 restoration, the functional regulation of several processes detected by our previous in vitro and in vivo studies is related to a transcriptional regulation of corresponding gene sets. Furthermore, interestingly, the results obtained with the in vitro model seem to reflect what we observed in TCGA-PTC tumors stratified by different TIMP3 expression levels, and suggest a direct link between TIMP3 expression and transcriptional modulation of specific gene sets.

## Conclusions

In summary, our results highlight that the expression level of TIMP3 in thyroid tumor cells is likely to transcriptionally regulate several processes including inflammation and tumor immune infiltration. Specifically, our data unveil the different gene sets/functions correlated with TIMP3 loss. How the level of TIMP3 protein modulates gene expression, and the mediators involved in this process, as well as possible impact of TIMP3 loss on treatment response in preclinical and clinical settings remain to be investigated, and warrant further studies. Overall, this study provides a broader view on the onco-suppressive role of TIMP3 in thyroid cancer, useful for the design of therapeutic approaches based on TIMP3 restoration.

## Materials and methods

### TCGA data and CIBERSORT analysis

RNA-Sequencing by Expectation Maximization (RSEM) counts data were obtained in 486 PTC cases with clinical and molecular data freely accessible from Cancer Genome Atlas (TCGA) consortium [4]. RSEM of 20,531 Entrez ID annotated genes were compared in IV versus I TIMP3 quartile (122 samples in each group), by using DeSeq2 package in R environment (version 4.0.0). Genes under 10 total read counts were filtered out as a pre-filtering step. Out of 18,845 genes with non-zero total read counts, 1660 genes (949 up-regulated and 711 down-regulated) were differentially expressed at FDR < 1%, showing a FC of at least 2 in absolute value, between the two groups (underlined genes in Additional file 11: Table S10). Normalized expression levels (Fragments per Kilobase Million, FPKM, values) of 26,572 annotated unique genes, available in 500 TCGA PTC cases, were subjected to CIBERSORT analysis. Cell fractions detection analysis was performed on the base of LM22 gene signature matrix, represented by 547 genes distinguishing 22 human hematopoietic cell phenotypes, including T-cell types, naïve and memory B cells, plasma cells, natural killer cells and myeloid subsets [43]. Deconvolution results of LM22 signature were compared between the extreme TIMP3 quartiles by Wilcoxon test. The resulting p-values were adjusted by Benjamini-Hochberg (BH) method.

### Cell lines

NIM1-EV and NIM-T23 stable clones, established as previously described [30], were maintained in DMEM medium containing 10% FCS, 2 mM glutamine and 100 U/ml penicillin/streptomycin with the addition of 400 μg/ml G418 (Lifetechnologies, Invitrogen, Carlsbad, CA, USA).

### Real-time PCR

RNA extraction was performed with Nucleospin RNA purification kit (MACHEREY–NAGEL GmbH & Co. KG, Düren, Germany) according to the manufacturer’s instructions. 1 μg of RNA was retrotranscribed using SuperscriptIII (Invitrogen, Carlsbad, CA, USA) following the manufacturer’s instructions. Expression of inflammatory related genes was analyzed by a customized TaqMan Low Density Array (Applied Biosystems) using 2 ng of retrotranscribed RNA as template. Data analysis was performed using SDS (Sequence Detection System) 2.4 software.

### Global gene expression profiling

Global gene expression profiles were obtained on total RNA samples of NIM1-T23 and NIM1-EV triplicate samples by means of GeneChip^®^Human Gene 2.0 ST arrays (ThermoFisher), according to the manufacturer's instructions. Normalized gene expression values and Brainarray (version 20) custom annotation were obtained as previously described [44].

Supervised analysis was carried out by comparing NIM1-T23 versus NIM1-EV triplicates using the Rank Product method [43] in R environment. The list of differentially expressed genes was selected by a cut-off of 1% on the Percentage of False Positive (PFP) and at least 2 as absolute value of FC. Global gene expression data are available under Gene Expression Omnibus (GEO) accession number GSE206915.

### Annotation enrichment analysis

Functional analysis with clusterProfiler [46, 47] was performed in R environment on DE gene lists, using enrichGO (q-value = 0.10) and enrichKEGG (q-value = 0.25) functions. Redundancy of enriched GO terms was removed (default cut-off = 0.07) on the enrichGO output. The top selected GO-BP terms and KEGG pathway gene sets were represented by cnetplot.

### GSEA

Gene Set Enrichment Analysis (GSEA) was performed on 8010 gene list ranked according to log2 fold change value (Additional file 11: Table S10). Global expression values were used to compare NIM1-T23 versus NIM1-EV triplicates, by applying gene set permutations.

Default analysis conditions were applied on Hallmark collection (version 7.2) gene sets (15–500 genes). Significant gene sets were selected on the base of nominal p-value < 0.05 and FDR q-value < 5%.

### Methylation analysis and miRNA correlation

Level 3 methylation beta values from Illumina Human Methylation 450 array were obtained for TCGA–THCA cohort by using the GDC Data Transfer Tool on GDC data portal (https://portal.gdc.cancer.gov/). In order to evaluate the methylation status of TIMP3 promoter, the average beta-value of four methylation probes annotated as CpG island around the Transcription Starting Site (− 297 to 364 nt, distance range from TSS) was considered in each sample. Global methylation levels were then calculated as the average beta-value of all ten methylation probes, including also the ones annotated in more distant regions (N-shore and S-shelf CpG islands) to TSS (− 1363 to 2616 nt, distance range from TSS). Box plot distributions of average beta-values in TIMP3 were evaluated in 121 quartile I and in 122 quartile IV PTC cases, using ggplot2 R package. The significance of differences between groups was estimated by Wilcoxon rank sum test with continuity correction. Spearman correlation coefficient rho was calculated to measure the correlation between TIMP3 expression and methylation levels, in quartile I and IV, respectively, setting “less” (negative association) as the alternative hypothesis to test.

Mirbase 21 mirna quantification files were obtained from miRNA sequencing data of TCGA-THCA cohort, by using the GDC Data Transfer Tool on GDC data portal (https://portal.gdc.cancer.gov/). Reads_per_million_miRNA_mapped values were extracted for hsa-mir-21, hsa-mir221, hsa-mir-222 and hsa-mir-373 selected miRNAs. Spearman correlation coefficient rho was calculated to measure the correlation between TIMP3 and hsa-miRNAs expression levels, in quartile I and IV, including 122 PTC cases, respectively. Benjamini–Hochberg procedure (BH) was applied for multiple test correction.

### Survival analysis

Kaplan–Meier curves were obtained on available Overall Survival (OS) data of 106 TIMP3 quartile I and 100 TIMP3 quartile IV PTC cases in TCGA cohort, by survival and survminer R packages. Log-Rank test p-value was calculated to measure the difference between survival curves. Data manipulation and all the statistical tests were performed in R environment (version 4.1.2).

## Supplementary Information

**Additional file 1: Table S1.** Correlation between TIMP3 expression and TSS/promoter or global methylation levels in quartile I (121 PTCs) and IV (122 PTCs), respectively, from TCGA cohort. Spearman correlation coefficient rho and p-value are reported. Benjamini–Hochberg procedure (BH) was applied for multiple test correction (p-val_adj). Significant results are reported in red.

**Additional file 2: Table S2.** Correlation between TIMP3 and selected hsa-miRNAs expression levels in TIMP3 quartile I and IV (122 PTC cases in each group). Spearman correlation coefficient rho and p-value are reported. Benjamini–Hochberg procedure (BH) was applied for multiple test correction (p-val_adj). Significant results are reported in red.

**Additional file 3: Table S3.** Clinical and molecular data of TCGA-PTC tumors in I and IV TIMP3 quartiles. Fisher's exact test measuring the association between TIMP3 expression levels and the occurrence of BRAFV600E mutation and patient characteristics in PTC cases of TCGA cohort for which molecular and clinical information were available. Benjamini–Hochberg procedure (BH) was applied for multiple test correction (adj_p-value). Samples are stratified in I and IV quartiles according to TIMP3 expression levels.

**Additional file 4: Figure S1.** Kaplan–Meier survival curves on OS data available in 206 PTC cases stratified according to TIMP3 quartile I and IV in TCGA-THCA cohort. Log-rank test p-value is reported.

**Additional file 5: Table S4.** Hallmark gene sets significantly enriched in IV versus I TIMP3 quartile. Gene sets were selected on nominal p-value < 0.05 and FDR q-value < 5%. Size and Normalized Enrichment Score (NES) are also reported for each gene set. Up and down- regulated gene sets are ordered according to NES. Red asterisk indicate Hallmark gene sets significantly enriched also in NIM-T23 versus NIM-EV clones.

**Additional file 6: Table S5.** GO functional enriched terms in IV versus I TIMP3 quartile. Details of the top 20 significantly enriched GO-BP terms in TIMP3-high versus TIMP3-low TCGA cases by cluster Profiler analysis on 1660 differentially expressed genes with at least 2 absolute FC.

**Additional file 7: Table S6.** KEGG functional enriched terms in IV versus I TIMP3 quartile. Details of the top 25 significantly enriched KEGG gene sets in TIMP3-high versus TIMP3-low TCGA cases by cluster Profiler analysis on 1660 differentially expressed genes with at least 2 absolute FC.

**Additional file 8: Table S7.** Hallmark gene sets significantly enriched in NIM1-T23 versus NIM1-EV clones. Gene sets were selected on nominal p-value < 0.05 and FDR q-value < 5%. Size and Normalized Enrichment Score (NES) are also reported for each gene set. Gene sets are ordered according to NES. Red asterisk indicate Hallmark gene sets significantly enriched also in IV versus I TIMP3 tumors quartiles.

**Additional file 9: Table S8.** Supervised gene expression analysis results in NIM1-T23 versus NIM1-EV clones. List of 290 differentially expressed genes in NIM1-T23 (expressing a high level of TIMP3 protein) and NIM1-EV (control empty vector) triplicates by Rank product at PFP < 1% and FC > 2 or < − 2. Up- and down-regulated genes are ordered according to absolute value of FC.

**Additional file 10: Table S9.** GO functional enriched terms in NIM1-T23 versus NIM1-EV clones. Details of the top 30 significantly enriched GO-BP terms in NIM1-T23 clone compared to NIM1-EV control by cluster Profiler analysis on 290 differentially expressed genes.

**Additional file 11: Table S10.** Differential gene expression analysis in IV versus I TIMP3 quartile. List of 8010 differentially expressed genes by DeSeq2 analysis in IV versus I TIMP3 quartile at FDR < 1%. Up- (3853/8010, 48%) and down-regulated (4157/8010, 52%) genes are ordered according to fold change (FC) value. Gene symbols of 1660 genes (949/1660, 57% up-regulated and 711/1660, 43% down-regulated) showing a FC of at least 2 are underlined.

## Data Availability

The PTC datasets analyzed during the current study were downloaded from the TCGA data portal (http://www.cbioportal.org/). Global gene expression data on TIMP3-restored NIM1 cells are available under Gene Expression Omnibus (GEO) accession number GSE206915.

## Abbreviations

PTC: Papillary thyroid cancer
FTC: Follicular thyroid cancer
ATC: Anaplastic thyroid cancer
PDTC: Poorly differentiated thyroid cancer
TIMP3: Tissue inhibitor of metalloproteinase-3
MMPs: Matrix metalloproteinases
ECM: Extracellular matrix
TAMs: Tumor associated macrophages
TCGA: The cancer genome atlas
GSEA: Gene set enrichment analysis
RSEM: RNA-sequencing by expectation maximization
EV: Empty vector
FC: Fold change
FDR: False discovery rate
NES: Normalized enrichment score
GO: Gene ontology
KEGG: Kyoto encyclopedia of genes and genomes
CIBERSORT: Cell-type identification by estimating relative subsets of RNA transcripts
