# Supplementary figures and images for: Transcriptomic landscape of TIMP3 oncosuppressor activity in thyroid carcinoma
Source: Cancer Cell Int. 2022 Dec 12;22:400. doi: 10.1186/s12935-022-02811-8 (PMC9743531; doi:10.1186/s12935-022-02811-8)

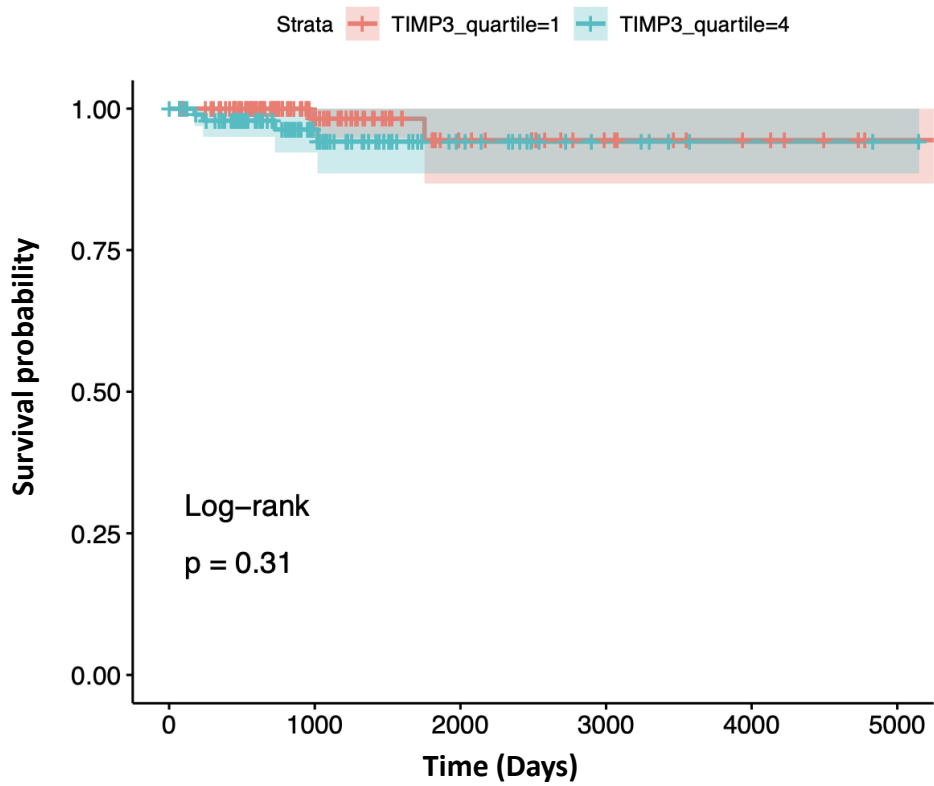

Supplement: Supplementary file 4 — Additional file 4: Figure S1. Kaplan–Meier survival curves on OS data available in 206 PTC cases stratified according to TIMP3 quartile I and IV in TCGA-THCA cohort. Log-rank test p-value is reported. [file 12935_2022_2811_MOESM4_ESM.pdf]
